# The Mechanisms of the Herbal Components of CRSAS on HK-2 Cells in a Hypoxia/Reoxygenation Model Based on Network Pharmacology

**DOI:** 10.1155/2020/5352490

**Published:** 2020-04-13

**Authors:** Naijing Ye, Dengpiao Xie, Bing Yang, Mingquan Li

**Affiliations:** ^1^Hospital of Chengdu University of Traditional Chinese Medicine, Chengdu, Sichuan, China; ^2^Sichuan 2nd Hospital of Traditional Chinese Medicine, Chengdu, Sichuan, China

## Abstract

**Background:**

Acute kidney injury is a global problem, which brings a great burden to the society and family. The component of rhubarb, *Salvia miltiorrhiza*, *Astragalus membranaceus*, and safflower (CRSAS) has been proved as an useful agent to treat acute kidney injury (AKI) patients in China.

**Objective:**

To assess the effect of CRSAS on human renal tubular epithelial cells (HK-2) after the hypoxia/reoxygenation (H/R) and investigate the potential mechanisms.

**Methods:**

Network pharmacology was used to predict the potential pathways shared by CRSAS and AKI. Cell counting kit-8 (CCK-8) was used to assess the HK-2 vitality. Apoptosis of HK-2 cells was detected by carboxyfluorescein succinimidyl ester/propidium iodide (CFSF/PI) staining. Expression of GRP78, CHOP, caspase-3, and Bax was detected by western blot and quantitative real-time RT-PCR.

**Result:**

CRSAS and AKI shared the endoplasmic reticulum stress (ERS) pathway based on network pharmacology analysis. CRSAS increases the vitality of HK-2 cells and reduces the apoptosis of HK-2 cells induced by H/R injury. The expression of GRP78 and CHOP in CRSAS groups was lower than that of control groups.

**Conclusions:**

H/R can induce HK-2 cell apoptosis and ERS. CRSAS can reduce HK-2 cell apoptosis by inhibiting the ERS. Therefore, CRSAS might be able to treat kidney disease due to I/R injury. Animal experiment should be done to further prove our finding.

## 1. Introduction

Acute kidney injury (AKI) is a common disease that often leads to high risk of morbidity and mortality. Although some AKIs can be reversed, the risk is high for AKI patients to develop chronic kidney disease [1]. There are many factors that affect the development and prognosis of AKI. Ischemia/reperfusion (I/R) injury is a common cause of AKI [2]. There are several pathophysiological processes that may lead to renal injury after I/R. When the kidney suffers from hypoxia and reoxygenation, which can lead to endoplasmic reticulum stress (ERS), reactive oxygen species (ROS) production, and inflammation in kidney cells, especially in human kidney-2 (HK-2; human renal proximal tubular epithelial cells), and cause HK-2 cell injury and apoptosis [3]. Misfolded proteins can accumulate in the endoplasmic reticulum of HK-2 cells after I/R, which leads to cell injury. Gentamicin causes toxicity to kidneys, and it is associated with ERS, inflammation, and apoptosis. Improved renal function and attenuated ERS occurred when gentamicin-induced nephrotoxicity was treated with atorvastatin [4]. Therefore, inhibiting ERS can improve AKI.

The Chinese herbal medicine components of Chinese rhubarb (*Rheum palmatum*), salvia (*Salvia miltiorrhiza*), astragalus (*Astragalus membranaceus*), and safflower (*Carthamus tinctorius*) (CRSAS) are used clinically for the treatment of AKI in China. Previous studies have shown that CRSAS is effective in treating patients with AKI. Basic experiments showed that CRSAS can alleviate kidney edema in rabbits and decrease renal tubular degeneration and necrosis in rabbits with AKI [5]. Furthermore, it can promote the regeneration and repair of necrotic cells in renal tubules. However, the mechanism of CRSAS in the improvement of AKI is unclear. In order to explore the underlying mechanism, network pharmacology was used herein to predict the targets of CRSAS and AKI. Enrichment analysis was performed based on targets to determine potential pathways, and related experiments were then performed to verify the results of prediction.

Chinese herbs contain multiactive components and affect diseases with multiple targets and pathways. Using conventional experimental methods to accurately determine the mechanism of Chinese herbs is ineffective [6]. Therefore, a new research method is required to effectively study the mechanism of Chinese herbs. Network pharmacology is suitable because examination can be performed on the multicomponent interactions with multiple targets (genes or proteins), and this process is analogous to how Chinese herbs act on diseases [7]. Our network pharmacology study mechanism includes the following main steps: determining the components of the Chinese herb; analyzing the components by druglikeness, Caco-2 permeability, and bioavailability; collecting targets of the components and the disease; determining the protein-protein interaction (PPI) of targets of components and the disease; searching the core target shared by components and the disease; and performing enrichment analysis of the core targets. The use of this method will enable us to have a clearer understanding of the effect of CRSAS on AKI based on network pharmacology approaches.

## 2. Methods

### 2.1. Network Pharmacology-Based Prediction of the Potential Pathway of CRSAS on AKI

#### 2.1.1. Searching Targets in CRSAS and AKI

The components of CRSAS were located in the Traditional Chinese Medicine Integrated Database (TCMID) (http://www.megabionet.org/tcmid/). To select the active components, the parameters of druglikeness >0.18, Caco-2 permeability >0.4, and bioavailability >30% were used. The targets of the components were also located in the TCMID. PharmGkb (http://www.pharmgkb.org) and GAD (https://geneticassociationdb.nih.gov/) were used to collect the targets of AKI.

#### 2.1.2. Protein-Protein Interaction of Targets

The network analyzer software Cytoscape [8] was used to create the PPI network of the targets of CRSAS and AKI (http://cytoscape.org/).

#### 2.1.3. Searching the Core Targets Shared by CRSAS and AKI

Cores targets shared by CRSAS and AKI were collected by the CytoNCA plugin.

#### 2.1.4. Pathway Enrichment

The data were examined using the Database for Annotation, Visualization, and Integrated Discovery (DAVID) (https://david.ncifcrf.gov/), which is an annotation tool involving Gene Ontology and Kyoto Encyclopedia of Genes and Genomes (KEGG) pathway enrichment analysis. OmicShare tools (http://www.omicshare.com/tools) was used to make a bubble chart.

### 2.2. Experiment to Verify the Network Pharmacology Predictions

#### 2.2.1. Preparation of CRSAS-Containing Serum

Chinese medicine decoction may have different pH or osmotic pressure from internal environment. These factors could influence cells when we culture cells. In order to remove these factors, we used drug-containing serum to treat cells. Six male adult New Zealand white rabbits, specific pathogen-free (SPF) grade, were purchased from Chengdu Dashuo Experimental Animal Co., Ltd., license no. SCXK2013-14. Animals were housed in the animal facilities of the Affiliated Hospital of Chengdu University of Traditional Chinese Medicine. The six male rabbits were randomly divided into the phosphate-buffered saline (PBS) group (the model group), who received treatment with PBS, and the CRSAS group, who received treatment with the herbal mixture. The drug dose was converted from human body weight to animal surface area, and the dose was 9.617 ml/kg daily via enema. PBS-containing serum and CRSAS-containing serum samples were obtained after administering the enemas to the rabbits on the fourth day.

#### 2.2.2. Cell Culture

HK-2 cells were purchased from Shanghai Biotechnology Co., Ltd. HK-2 cells were cultured in Dulbecco's modified Eagle's medium (DMEM) with 10% fetal bovine serum and then placed in humidified incubators at 37°C with 5% CO_2_. Trypsin was used for subculture. HK-2 cells were passaged 1 time every 3–5 days.

#### 2.2.3. Cell Hypoxia/Reoxygenation Test and Grouping of Cells

HK-2 cells were seeded in 24-well plates at a density of 2 × 10^4^–5 × 10^4^ cells per well for 24 h. HK-2 cells were cultured under hypoxic conditions in a medium without nutrients for 5 h. Drug-containing serum was added to HK-2 cell culture plates after reoxygenation. HK-2 cells were divided into three groups: the hypoxia/reoxygenation (H/R) model group, the CRSAS group (H/R model with administration of drug-containing serum), and the control group (without establishing an H/R model). The related index of each group was assessed at 4 h and 12 h after establishing the H/R model.

#### 2.2.4. Drugs and Reagents

CRSAS was supplied by Hainan Tianyuan Pharmaceutical Factory, Z46020083. SDS and TRIzol reagent were purchased from Amresco (USA). SuperSignal West Dura Stable Peroxide Buffer, SuperSignal West Dura Luminol Enhancer Solution, and PageRuler™ Prestained Protein Ladder were purchased from Thermo (USA). Cell Counting Kit-8 (CCK-8) was purchased from Dongren Chemical Technologies (Shanghai, China). Carboxyfluorescein succinimidyl ester (CFSE, 400x) and propidium iodide (PI, 400x) were purchased from Sigma (USA). Pancreatin (without EDTA), fetal bovine serum (FBS), and Dulbecco's modified Eagle's medium/Nutrient Mixture F-12 (DMEM/F-12) were purchased from Gibco (USA). An FITC Annexin V Apoptosis Detection Kit was purchased from Becton Dickinson (USA). Tris-HCl was purchased from Solarbio (Beijing, China).

#### 2.2.5. Cell Vitality Assay

The CCK-8 assay was used to detect the vitality of HK-2 cells. HK-2 cells (5 × 10^3^ cells per well) were plated onto 96-well plates. CCK-8 solution (10 *μ*L per well) was added at 4 h and 12 h after establishing the H/R model, and the cells were incubated at 37°C for 1 h. The absorbance at 450 nm was read with a microplate reader.

#### 2.2.6. CFSE/PI Staining Assay

Cell apoptosis was assessed by CFSE/PI staining. The cells were subcultured at a density of 2 × 10^4^–5 × 10^4^ cells/well in a 24-well plate. The cells were incubated with 500 *μ*L-5 *μ*M CFSE working liquid. The CFSE stain was removed, and then, HK-2 cells were washed with PBS. At each specific time, 200 *μ*L PI working liquid was added in the dark to each well for 5 min. The viable HK-2 cells emitted green light under a fluorescence microscope, and apoptotic HK-2 cells emitted red light.

#### 2.2.7. Quantitation of GRP78, CHOP, ERO1-A, Caspase-3, and Bax mRNA by Real-Time RT-PCR

According to the manufacturer's protocol, total RNA was separated using TRIzol reagent and reverse transcribed to first-strand cDNA with Moloney murine leukemia virus (MMLV) reverse transcriptase. The primer sequences are as follows:

  GAPDH: (forward primer) GGAGCGAGATCCCTC-CAAAAT, (reverse primer) GGCTGTTGTCATACTT-CTCATGG
  GRP78: (forward primer) CATCACGCCGTCCTAT-GTCG, (reverse primer) CGTCAAAGACCGTGTTC-TCG
  CHOP: (forward primer) GGAAACAGAGTGGTCATTCCC, (reverse primer) CTGCTTGAGCCGTTCA-TTCTC
  Caspase-3: (forward primer) CATGGAAGCGAATCAATGGAC, (reverse primer) CTGTACCAGACCGAGATGTCA
  Bax: (forward primer) CCCGAGAGGTCTTTTTCCGAG, (reverse primer) CCAGCCCATGATGGTT-CTGAT

Real-time RT-PCR amplification was performed with the use of SYBR® Green Master Mix and an Applied Biosystems 7500 Real-time PCR Detection System. All samples were run in triplicate and underwent 40 amplification cycles. The thermocycling conditions were 95°C for 10 sec, 60°C for 30 sec, and 72°C for 20 sec. Bio-Rad CFX 2.1 management software was utilized for data analysis. Gene expression in the PBS group was used as an internal reference. The differences in mRNA expression levels between samples were determined using the 2^−ΔΔ Cq^ relative quantification method [2].

#### 2.2.8. Western Blotting for GRP78, CHOP, Caspase-3, and Bax Protein

Cells were solubilized with lysis buffer, centrifuged, and the supernatants were removed. Proteins were separated by sodium dodecyl sulfate-polyacrylamide gel electrophoresis (SDS-PAGE) and transferred to a polyvinylidene difluoride (PVDF) membrane. Then, the membranes were blocked with 5% skim milk and Tris-buffered saline (TBS) containing 0.1% Tween-20 (TBST). A primary antibody was diluted with PBST (containing 0.1% Tween-20 PBS solution) in accordance with the antibody instructions and incubated for 1.5 h at room temperature or 4°C. Then, the blots were rinsed with PBST for 5 minutes and washed 3 times. A secondary antibody was diluted with PBST according to the antibody instructions, and the secondary antibody was incubated for 1 h at room temperature or 37°C. Then, the blots were rinsed 3 times with PBST after incubation to remove the excess secondary antibody. *β*-Actin was used as an internal reference, and Quantity One software was used to obtain the experimental data from the western blots. The relative expression level of GRP78, CHOP, and caspase-3 is conveyed by the ratio of protein to *β*-actin.

## 3. Statistical Analysis

The data are expressed as the mean ± standard deviation. Two sets of independent *t*-tests were used to compare the results of the two groups. The results of three or more groups were compared by one-way ANOVA. All statistical analyses were performed using SPSS 21.0; a significant difference was defined as *P* < 0.05.

## 4. Results

### 4.1. Prediction Pathways Obtained for CRSAS on AKI

**Figure 1:**
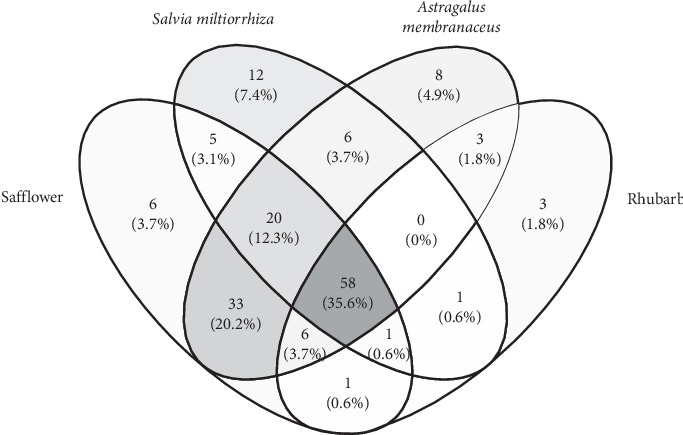
The target number for rhubarb (*Rheum palmatum*), Chinese salvia (*Salvia miltiorrhiza*), astragalus (*Astragalus membranaceus*), and safflower (*Carthamus tinctorius*).

**Figure 2:**
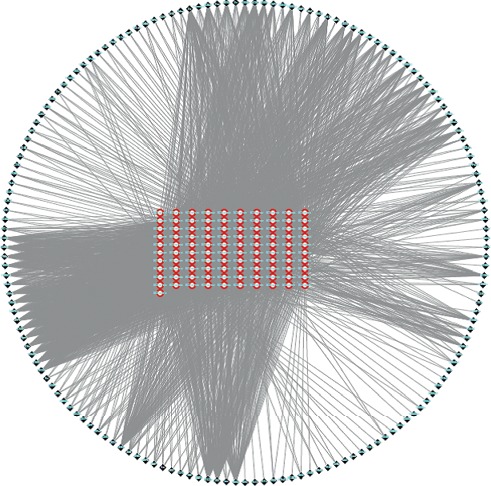
The network of targets and components of CRSAS. Red points represent active components. Black points in the outer layer represent targets. CRSAS, compound of rhubarb, *Salvia miltiorrhiza*, *Astragalus membranaceus*, and safflower.

**Figure 3:**
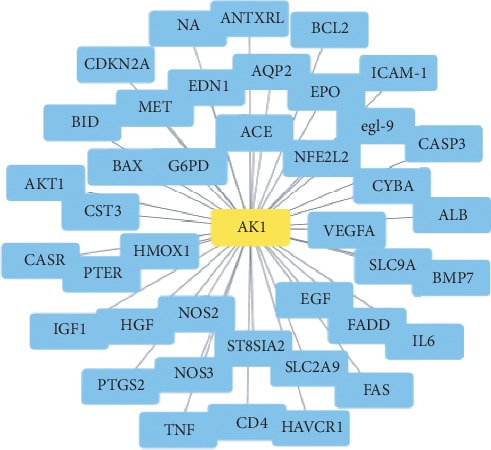
The network of targets and AKI. AKI, acute kidney injury.

**Figure 4:**
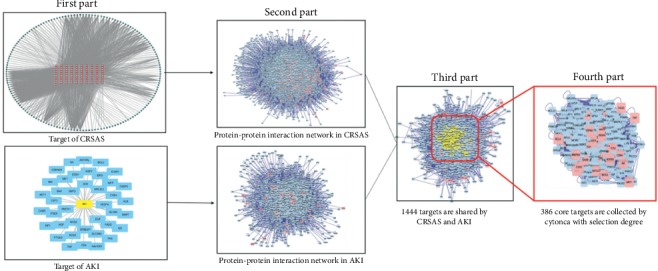
The flow of searching core targets shared by CRSAS and AKI. The first part searched the targets of CRSAS and AKI. The second part searched the potential targets by the protein-protein interaction network. The third part found the targets shared by CRSAS and AKI. The fourth part found the most important targets shared by CRSAS and AKI. CRSAS, compound containing rhubarb, salvia, astragalus, and safflower; AKI, acute kidney injury.

CRSAS has 121 active components and 163 targets. The targets of rhubarb, salvia, astragalus, and safflower are shown in Supplement 1 and Figure 1. In Figure 2, the red points represent active components and the outer layer black points represent targets. One component can aim at different targets, and one target can be aimed by different components. These active components might treat AKI through these targets. There were 40 targets that were found in AKI in Figure 3. Proteins regulate the expression of other proteins. So, 163 targets in CRSAS and 40 targets in AKI might influence other targets, in order to search potential targets. PPI network analysis was used to search potential targets. PPI network analysis found 1813 targets and 6937 targets in CRSAS and AKI, respectively. The shared targets might be the genes that CRSAS treats AKI. Therefore, we used Software CytoNCA to find core 386 target shared by CRSAS and AKI. The flow chart in Figure 4 shows the search process used to obtain core targets.

**Figure 5:**
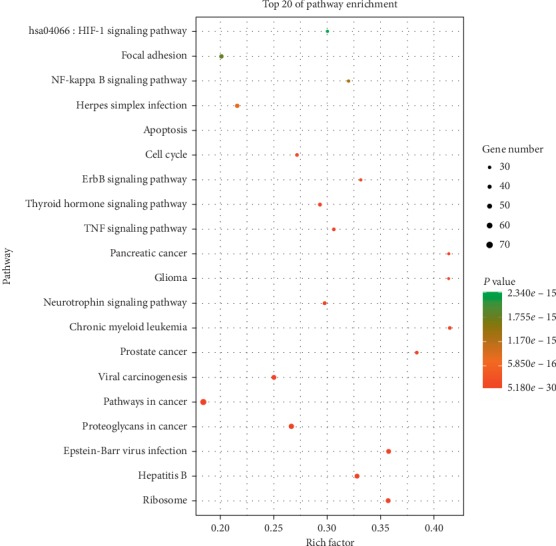
Pathway enrichment analysis of core targets of CRSAS, as shown in the bubble chart. CRSAS, compound containing rhubarb, salvia, astragalus, and safflower; AKI, acute kidney injury.

The 386 important genes were analyzed by OmicShare tools to find the related pathways shown in Figure 5. According to these pathways, we assumed that apoptosis has a closer connection with AKI. Therefore, we further analyzed the apoptosis pathway by KEGG and based on results of KEGG, and we found that CRSAS might treat AKI through endoplasmic reticulum stress.

### 4.2. Experimental Pharmacology Results

#### 4.2.1. CRSAS Increased HK-2 Cell Vitality and Inhibited Apoptosis of HK-2 Cells

**Figure 6:**
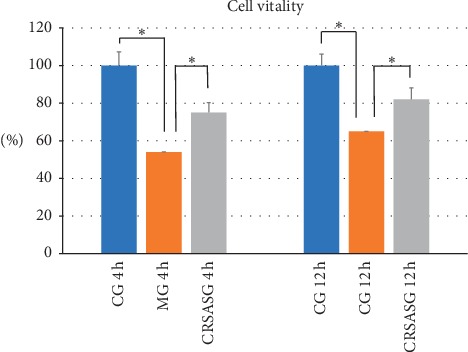
HK-2 cell vitality determined by CCK-8. HK-2 cells, human renal tubular epithelial cells; CCK-8, Cell Counting Kit-8; CRSASG, compound of rhubarb, salvia, astragalus, and safflower group.

**Figure 7:**
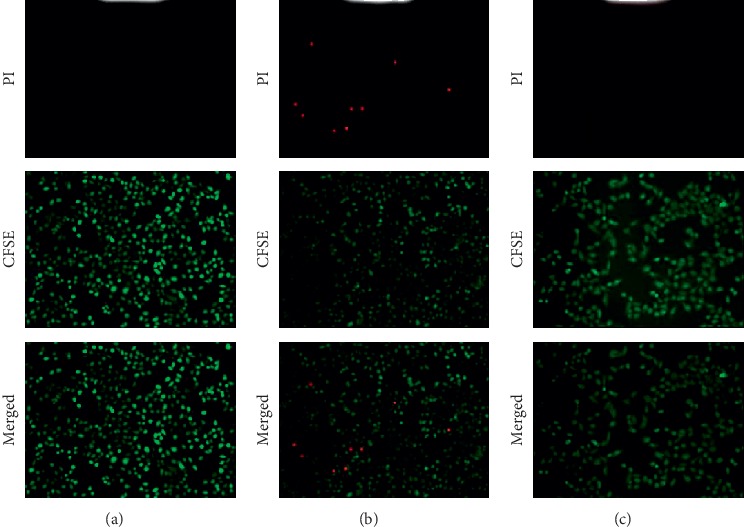
Apoptosis of HK-2 cells detected by CFSE/PI at 4 h after establishing the H/R model. HK-2 cell, human renal tubular epithelial cell; CFSE, carboxyfluorescein succinimidyl amino ester; PI, propidium iodide; CRSASG, compound of rhubarb, salvia, astragalus, and safflower group. (a) CG 4 h, (b) MG 4 h, and (c) CRSASG 4 h.

**Figure 8:**
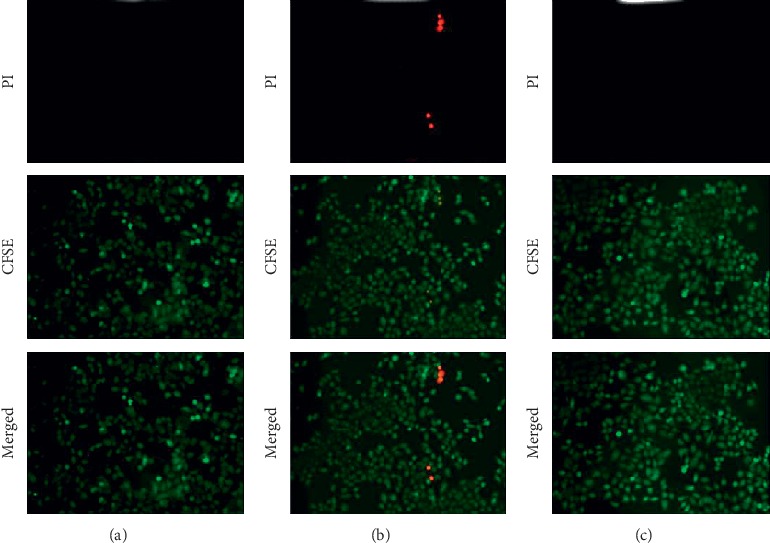
Apoptosis of HK-2 cells detected by CFSE/PI at 12 h after establishing the H/R model. (a) CG 12 h, (b) CG 12 h, and (c) CRSASG 12 h.

**Figure 9:**
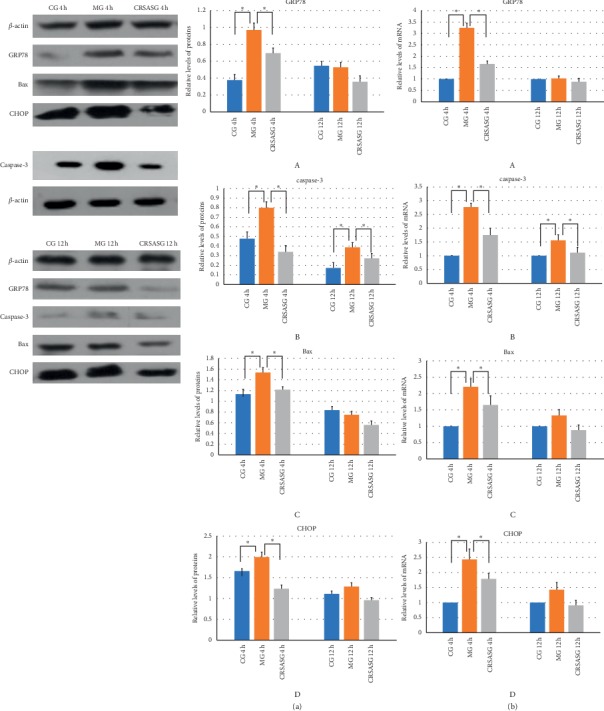
(a) Time-response study on the HK-2 protein expression of (A) GRP78, (B) caspase-3, (C) Bax, and (D) CHOP. (b) Time-response study on the HK-2 mRNA expression of (A) GRP78, (B) caspase-3, (C) Bax, and (D) CHOP. CRSASG, compound of rhubarb, salvia, astragalus, and safflower group.

The CCK-8 assay as shown in Figure 6 demonstrated that the vitality of HK-2 cells significantly decreased after establishing the H/R model. Figure 6 also shows that CRSAS significantly increased HK-2 cell vitality compared with the model group, as the CFSE staining indicated that the number of apoptotic HK-2 cells increased after establishing the H/R model. Figures 7 and 8 show that CRSAS reduced the number of apoptotic HK-2 cells. The expression of proapoptotic caspase-3 and Bax represents the activation of the apoptotic pathway in HK-2 cells. We found that the expression of caspase-3 and Bax was significantly increased after establishing the H/R model, as shown in Figures 9(c), 9(d), 9(e), and 9(f). CRSAS inhibited the expression of caspase-3 and Bax as compared with the model group, as shown in Figures 9(c), 9(d), 9(e), and 9(f). The above results demonstrated that CRSAS can alleviate H/R-induced HK-2 cell injury and inhibit the apoptotic pathway activation.

#### 4.2.2. ERS Was Inhibited by CRSA

In Figures 9(a) and 9(b), the H/R model increased the expression level of GRP78 compared with the control group at 4 h (*P* < 0.05), and the expression of GRP78 returned to normal at 12 h. CRSAS inhibited the expression level of GRP78 at 4 h (*P* < 0.05), as shown in Figures 9(a) and 9(b). In Figures 9(g) and 9(h), the H/R model increased the expression level of CHOP compared with the control group at 4 h (*P* < 0.05). CRSAS inhibited the expression level of CHOP at 4 h (*P* < 0.05), and the expression of CHOP returned to normal at 12 h, as seen in Figures 9(g) and 9(h). The above results demonstrated that H/R can activate ERS, which can lead to apoptosis of HK-2 cells. CRSAS can inhibit the CHOP pathway, which is downstream of ERS and inhibits the apoptotic pathway.

## 5. Discussion

Network pharmacology is a powerful tool that can explain the function of a complicated biological system [9]. Recently, it has been widely used to explore the pharmacological mechanisms of Chinese herbs, which has enabled it to be proved in an efficient way. There are many network pharmacology studies that have successfully uncovered the underlying mechanism of TCM in treating diseases. For example, Lieberthal and Nigam revealed the mechanism of the effect of Fufang Danshen on pain based on network pharmacology [10]. Bonventre and Yang uncovered the effect of Willd on colorectal cancer based on network pharmacology [11]. In TCM, a prescription often contains many Chinese herbs with the aim of creating synergistic effects between the different herbs. It is currently possible to prove such synergistic effects from combination therapy in TCM with the assistance of network pharmacology. Chatterjee et al. demonstrated that combination therapy can increase bioavailability and has a more optimal therapeutic effect as compared to therapy using only one herb [12].

In our study, we found that CRSAS has a total of 163 targets, and 58 (35.8%) targets were shared among the four herbs, which demonstrated that CRSAS has synergistic effects in treating disease. Enrichment analysis showed that the pathways of apoptosis, chronic myeloid leukemia, glioma, and pancreatic cancer have the highest enrichment factors. We assume that apoptosis could be the key pathway based on AKI. KEGG was further used to reveal the specific signaling pathway that led to cell apoptosis based on our targets, and the results showed that ERS might be associated with the effect of CRSAS on AKI.

A large, retrospective, population-based study showed that 11.6% of hospitalized adults from nine regional central hospitals developed AKI, with prerenal AKI in 51.8% of these AKI patients [13]. Therefore, ischemia plays a critical role in the development of AKI [14]. Several studies have shown that ischemia can cause apoptosis of HK-2 cells [15, 16]. Injury and death of renal tubular epithelial cells are characteristics of AKI induced by ischemia/reperfusion (I/R). Epithelial cells in the proximal tubule are commonly affected when ischemia occurs in the kidney [17]. Characteristic changes in histology in tubular cells include dilatation of the tubular lumen, loss or effacement of the tubular brush border, and formation of casts because of apoptosis [18]. Therefore, a decrease in tubular cell apoptosis reduces kidney injury and accelerates the recovery of kidney function.

The accumulation of unfolded proteins in the endoplasmic reticulum can result from metabolic dysfunction, hypoxia, and deprivation of energy, leading to ERS and an unfolded protein response (UPR) [19]. ERS plays an important role in the apoptosis of HK-2 cells induced by ischemic AKI [20]. Under normal conditions, GRP78 is combined with protein kinase RNA-like ER kinase (PERK), activating transcription factor 6 (ATF6), and inositol requiring enzyme 1 (IRE1). When the kidneys undergo hypoxia and deprivation of energy, GRP78 departs from PERK, ATF6, and IRE1 and causes upregulation of GRP78. Therefore, GRP78 is a hallmark of the initiation of ERS and a well-established biomarker of ERS.

Inhibition of ERS has demonstrated that it can alleviate AKI. Li et al. demonstrated that I/R-induced renal cell apoptosis and renal function loss were associated with ERS, and I/R-induced AKI was attenuated in mice when ERS was inhibited [21]. The activation of downstream molecule C/EBP homologous protein (CHOP) in ERS is important in the ERS-mediated apoptosis signaling pathway. CHOP can be activated by PERK, ATF6, and IRE1. CHOP is a proapoptotic gene, which leads to cell apoptosis and AKI. I/R-induced upregulation expression of CHOP leads to renal cell apoptosis and delays the recovery of kidney function [22]. Additionally, activation of CHOP causing Ca^2+^ released from the endoplasmic reticulum lumen aggravates ERS [14]. Elevated expression of CHOP results in the downregulation of the expression of the prosurvival molecule Bcl2 and increased expression of proapoptotic molecules caspase-3 and Bax [16]. Activation of endoplasmic reticulum oxidoreductase-1a (ERO1a) hyperoxidizes the endoplasmic reticulum and promotes cell apoptosis.

However, I/R injury also leads to oxidative stress and increases production of ROS, which leads to mitochondrial injury and ERS. ROS can lead to DNA fragmentation and cell apoptosis [15]. Chen et al. demonstrated that inhibiting ROS can attenuate apoptosis in kidney injury [23]. In our study, we did not assess the effect of CRSAS in oxidative stress. There is a possible mechanism that CRSAS can inhibit oxidative stress, thus attenuating ERS and apoptosis. However, our study focuses on ERS. In a future study, we would like to study the effect of CRSAS under oxidative stress.

Studies have demonstrated that the inhibition of overexpression of CHOP can decrease cell apoptosis and alleviate AKI. For example, when cisplatin was administered to mice to develop an AKI model, AKI was associated with activation of ERS, and kidney function increased and renal cell apoptosis decreased with the inhibition of expression of CHOP [17]. In another animal experiment, tunicamycin, a type of antibiotic, is known to lead to ERS and is used to induce AKI. Carlisle et al. used tunicamycin to induce overexpression of CHOP and renal tubular apoptosis in mice, and those abnormal changes were ameliorated with the treatment of 4-phenylbutyrate, an ERS inhibitor that can prevent misfolded protein aggregation [18]. In our study, we found that CRSAS inhibited GRP78, CHOP, and caspase-3 expression. Therefore, we speculate that the reduction of HK-2 cell apoptosis by CRSAS may be associated with the inhibition of ERS and downregulation of CHOP expression.

In conclusion, H/R can induce HK-2 cell apoptosis and ERS. CRSAS can reduce HK-2 cell apoptosis by inhibiting ERS and expression of CHOP. Therefore, CRSAS might be able to treat kidney disease due to I/R injury. Additional animal experiments should be performed to further prove our findings.

## Data Availability

All data are presented in the manuscript. Datasets used and/or analyzed in this study are available from the corresponding author upon reasonable request.

## Supplementary Materials

Supplementary Materials The targets of rhubarb, salvia, astragalus, and safflower are shown in Supplement 1.
