# Supplementary material for: The Mechanisms of the Herbal Components of CRSAS on HK-2 Cells in a Hypoxia/Reoxygenation Model Based on Network Pharmacology
Source: Evid Based Complement Alternat Med. 2020 Apr 13;2020:5352490. doi: 10.1155/2020/5352490 (PMC7174917; doi:10.1155/2020/5352490)
Supplement: Supplementary Materials — The targets of rhubarb, salvia, astragalus, and safflower are shown in Supplement 1. [file 5352490.f1.docx]

|  | **Chemical nubmer** | **Chemical name** |
| --- | --- | --- |
| Saflower | MOL000006 | luteolin |
|  | MOL000098 | quercetin |
|  | MOL000358 | beta-sitosterol |
|  | MOL000422 | kaempferol |
|  | MOL000449 | Stigmasterol |
|  | MOL000953 | CLR |
|  | MOL001771 | poriferast-5-en-3beta-ol |
|  | MOL002680 | Flavoxanthin |
|  | MOL002694 | 4-[(E)-4-(3,5-dimethoxy-4-oxo-1-cyclohexa-2,5-dienylidene)but-2-enylidene]-2,6-dimethoxycyclohexa-2,5-dien-1-one |
|  | MOL002695 | lignan |
|  | MOL002698 | lupeol-palmitate |
|  | MOL002706 | Phytoene |
|  | MOL002707 | phytofluene |
|  | MOL002710 | Pyrethrin II |
|  | MOL002712 | 6-Hydroxykaempferol |
|  | MOL002714 | baicalein |
|  | MOL002717 | qt_carthamone |
|  | MOL002719 | 6-Hydroxynaringenin |
|  | MOL002721 | quercetagetin |
|  | MOL002757 | 7,8-dimethyl-1H-pyrimido[5,6-g]quinoxaline-2,4-dione |
|  | MOL002773 | beta-carotene |
|  | MOL002776 | Baicalin |
| Salvia Miltiorrhiza | MOL000006 | luteolin |
|  | MOL000569 | digallate |
|  | MOL001601 | 1,2,5,6-tetrahydrotanshinone |
|  | MOL001659 | Poriferasterol |
|  | MOL001771 | poriferast-5-en-3beta-ol |
|  | MOL001942 | isoimperatorin |
|  | MOL002222 | sugiol |
|  | MOL002651 | Dehydrotanshinone II A |
|  | MOL002776 | Baicalin |
|  | MOL006824 | α-amyrin |
|  | MOL007036 | 5,6-dihydroxy-7-isopropyl-1,1-dimethyl-2,3-dihydrophenanthren-4-one |
|  | MOL007041 | 2-isopropyl-8-methylphenanthrene-3,4-dione |
|  | MOL007045 | 3α-hydroxytanshinoneⅡa |
|  | MOL007048 | (E)-3-[2-(3,4-dihydroxyphenyl)-7-hydroxy-benzofuran-4-yl]acrylic acid |
|  | MOL007049 | 4-methylenemiltirone |
|  | MOL007050 | 2-(4-hydroxy-3-methoxyphenyl)-5-(3-hydroxypropyl)-7-methoxy-3-benzofurancarboxaldehyde |
|  | MOL007051 | 6-o-syringyl-8-o-acetyl shanzhiside methyl ester |
|  | MOL007058 | formyltanshinone |
|  | MOL007059 | 3-beta-Hydroxymethyllenetanshiquinone |
|  | MOL007061 | Methylenetanshinquinone |
|  | MOL007063 | przewalskin a |
|  | MOL007064 | przewalskin b |
|  | MOL007068 | Przewaquinone B |
|  | MOL007069 | przewaquinone c |
|  | MOL007070 | (6S,7R)-6,7-dihydroxy-1,6-dimethyl-8,9-dihydro-7H-naphtho[8,7-g]benzofuran-10,11-dione |
|  | MOL007071 | przewaquinone f |
|  | MOL007077 | sclareol |
|  | MOL007079 | tanshinaldehyde |
|  | MOL007081 | Danshenol B |
|  | MOL007082 | Danshenol A |
|  | MOL007085 | Salvilenone |
|  | MOL007088 | cryptotanshinone |
|  | MOL007093 | dan-shexinkum d |
|  | MOL007094 | danshenspiroketallactone |
|  | MOL007098 | deoxyneocryptotanshinone |
|  | MOL007100 | dihydrotanshinlactone |
|  | MOL007101 | dihydrotanshinoneⅠ |
|  | MOL007105 | epidanshenspiroketallactone |
|  | MOL007107 | C09092 |
|  | MOL007108 | isocryptotanshi-none |
|  | MOL007111 | Isotanshinone II |
|  | MOL007115 | manool |
|  | MOL007118 | microstegiol |
|  | MOL007119 | miltionone Ⅰ |
|  | MOL007120 | miltionone Ⅱ |
|  | MOL007121 | miltipolone |
|  | MOL007122 | Miltirone |
|  | MOL007123 | miltirone Ⅱ |
|  | MOL007124 | neocryptotanshinone ii |
|  | MOL007125 | neocryptotanshinone |
|  | MOL007127 | 1-methyl-8,9-dihydro-7H-naphtho[5,6-g]benzofuran-6,10,11-trione |
|  | MOL007130 | prolithospermic acid |
|  | MOL007132 | (2R)-3-(3,4-dihydroxyphenyl)-2-[(Z)-3-(3,4-dihydroxyphenyl)acryloyl]oxy-propionic acid |
|  | MOL007140 | (Z)-3-[2-[(E)-2-(3,4-dihydroxyphenyl)vinyl]-3,4-dihydroxy-phenyl]acrylic acid |
|  | MOL007141 | salvianolic acid g |
|  | MOL007142 | salvianolic acid j |
|  | MOL007143 | salvilenone Ⅰ |
|  | MOL007145 | salviolone |
|  | MOL007149 | NSC 122421 |
|  | MOL007150 | (6S)-6-hydroxy-1-methyl-6-methylol-8,9-dihydro-7H-naphtho[8,7-g]benzofuran-10,11-quinone |
|  | MOL007151 | Tanshindiol B |
|  | MOL007152 | Przewaquinone E |
|  | MOL007154 | tanshinone iia |
|  | MOL007155 | (6S)-6-(hydroxymethyl)-1,6-dimethyl-8,9-dihydro-7H-naphtho[8,7-g]benzofuran-10,11-dione |
|  | MOL007156 | tanshinone Ⅵ |
| Astragalus Membranaceus | MOL000033 | (3S,8S,9S,10R,13R,14S,17R)-10,13-dimethyl-17-[(2R,5S)-5-propan-2-yloctan-2-yl]-2,3,4,7,8,9,11,12,14,15,16,17-dodecahydro-1H-cyclopenta[a]phenanthren-3-ol |
|  | MOL000098 | quercetin |
|  | MOL000211 | Mairin |
|  | MOL000239 | Jaranol |
|  | MOL000296 | hederagenin |
|  | MOL000354 | isorhamnetin |
|  | MOL000371 | 3,9-di-O-methylnissolin |
|  | MOL000374 | 5'-hydroxyiso-muronulatol-2',5'-di-O-glucoside |
|  | MOL000378 | 7-O-methylisomucronulatol |
|  | MOL000379 | 9,10-dimethoxypterocarpan-3-O-β-D-glucoside |
|  | MOL000380 | (6aR,11aR)-9,10-dimethoxy-6a,11a-dihydro-6H-benzofurano[3,2-c]chromen-3-ol |
|  | MOL000387 | Bifendate |
|  | MOL000392 | formononetin |
|  | MOL000398 | isoflavanone |
|  | MOL000417 | Calycosin |
|  | MOL000422 | kaempferol |
|  | MOL000433 | FA |
|  | MOL000438 | (3R)-3-(2-hydroxy-3,4-dimethoxyphenyl)chroman-7-ol |
|  | MOL000439 | isomucronulatol-7,2'-di-O-glucosiole |
|  | MOL000442 | 1,7-Dihydroxy-3,9-dimethoxy pterocarpene |
| Rhubarb | MOL000096 | (-)-catechin |
|  | MOL000358 | beta-sitosterol |
|  | MOL000471 | aloe-emodin |
|  | MOL000554 | gallic acid-3-O-(6'-O-galloyl)-glucoside |
|  | MOL002235 | EUPATIN |
|  | MOL002251 | Mutatochrome |
|  | MOL002259 | Physciondiglucoside |
|  | MOL002260 | Procyanidin B-5,3'-O-gallate |
|  | MOL002268 | rhein |
|  | MOL002276 | Sennoside E_qt |
|  | MOL002280 | Torachrysone-8-O-beta-D-(6'-oxayl)-glucoside |
|  | MOL002281 | Toralactone |
|  | MOL002288 | Emodin-1-O-beta-D-glucopyranoside |
|  | MOL002293 | Sennoside D_qt |
|  | MOL002297 | Daucosterol_qt |
|  | MOL002303 | palmidin A |
